# Real-world treatment patterns, outcomes, and economic costs by lines of therapy in patients with newly diagnosed multiple myeloma: a nationwide population-based cohort study in South Korea

**DOI:** 10.1007/s44313-025-00069-3

**Published:** 2025-04-15

**Authors:** Sung-Soo Park, YoungJu Park, Soomin Yoon, Doik Lee, Jihyeon Jeong, Kihyun Kim

**Affiliations:** 1 Department of Hematology, College of Medicine, Seoul St. Mary’s Hospital, The Catholic University of Korea, Seoul, Republic of Korea; 2 Medical Affairs, Janssen Korea Ltd, Seoul, Republic of Korea; 3 Real World Solutions, IQVIA Solutions Korea Ltd, Seoul, Republic of Korea; 4 Department of Medicine, Sungkyunkwan University School of Medicine, Samsung Medical Center, 81 Irwon-dong, Gangnam-gu, Seoul, 06351 Republic of Korea

**Keywords:** Multiple myeloma, Real-world data, Treatment pattern, Survival, Treatment cost

## Abstract

**Purpose:**

Given the notable increase in the incidence of multiple myeloma (MM) in Asia and advent of innovative treatments, this study aims to provide a comprehensive understanding of the treatment patterns, outcomes, and economic burden of MM across the lines of therapy (LOTs) in South Korea.

**Methods:**

This retrospective cohort study was conducted using data from the National Health Insurance claims data provided by the Health Insurance Review and Assessment Database. An identification algorithm was developed to detect the regimens and LOTs. Treatment patterns and outcomes were assessed as real-world treatment sequence, treatment duration (rwTD), time to next-line treatment (rwTTNT), and overall survival (rwOS). Economic burden was assessed as healthcare resource utilization (HCRU) and the cost incurred per person per month.

**Results:**

This study included 11,450 patients who were newly diagnosed with MM between January 2010 and December 2019. The observed real-world LOT patterns reflect the changes in South Korea’s reimbursement scheme. Mean treatment-free intervals decreased from 11.59 months (SD 16.23) to 2.77 months (SD 6.14) from the first LOT (LOT 1) to LOT 5. Median rwTTNT decreased from 26.61 months (95% CI: 25.69-27.57) to 12.40 months (95% CI: 11.55-13.49), and median rwOS decreased from 61.88 months (95% CI: 59.11-65.46) to 13.65 months (95% CI: 11.88-16.22). The HCRU and associated costs increased substantially with the LOT advancement.

**Conclusion:**

This large-scale observational study offers comprehensive insights into the real-world treatment of MM in South Korea. The study findings highlight the progressive nature of MM and increasing economic burden of advanced lines of treatment, underscoring the necessity for optimized treatment strategies.

**Supplementary Information:**

The online version contains supplementary material available at 10.1007/s44313-025-00069-3.

## Introduction

Multiple myeloma (MM) is one of the most frequently diagnosed hematological malignancies worldwide and is characterized by the proliferation of plasma cells in the bone marrow [1]. Globally, there were 155,688 MM cases in 2019 alone, with the number of deaths attributable to MM increasing from 51,862 in 1990 to 113,474 in 2019 [2]. Previous studies have documented that the highest incidence rates were observed in North America, Australia, New Zealand, and Europe, whereas the incidence rates were relatively low in Asia [3–5]. However, there is growing evidence that the number of MM cases in the Asian region is increasing rapidly, with an increase of 262% from 4,760 cases (95% confidence interval CI: 4,271-5,575) in 1990 to 17,218 cases (95% CI: 14,428-19,093) in 2016, highlighting the urgent need for an in-depth understanding of treatment patterns and outcomes in relevant clinical settings [6].

With the emergence of new treatment options and the combined use of existing therapies, followed by updates in treatment guidelines, the therapeutic landscape for MM has improved substantially over the past two decades [7, 8]. The introduction of novel agents, such as proteasome inhibitors (bortezomib, ixazomib, and carfilzomib), immunomodulatory drugs (thalidomide, lenalidomide, and pomalidomide), and monoclonal antibodies (daratumumab and elotuzumab), has made considerable progress, leading to improved treatment outcomes. To fully appreciate and leverage these advancements, it is important to understand patients' treatment journeys through current real-world experiences; however, there are limited up-to-date real-world data available on MM treatment in Asia. Moreover, although MM accounts for a small proportion of all cancer types, the costs related to its treatment and management are among the highest [9]. As MM progresses, patients undergo subsequent treatment, and treatment patterns and outcomes change considerably, accompanied by a substantial increase in the economic burden. It is crucial to explore the current treatment trends in line with an understanding of the associated medical costs to facilitate optimal choices in MM management.

This retrospective database cohort study utilized the claims data from Health Insurance Review and Assessment (HIRA) to contribute to better understanding of the treatment patterns, outcomes, and economic burden of MM across lines of therapies (LOT) in South Korea.

## Materials and methods

### Study design and data source

A nationwide population-based retrospective cohort study was performed using national claims data provided by the HIRA in South Korea. The HIRA database covers approximately 98% of the Korean population and contains extensive information on healthcare utilization in inpatient and outpatient settings [10]. All information on adult patients (≥ 19 years) who had a confirmed diagnosis of MM from 1 January 2010 to 31 December 2019 (i.e., the index period) was obtained from the HIRA database. Eligible patients were selected using the International Classification of Diseases 10th Edition (ICD-10) codes and HIRA-reimbursed drug and procedure codes. The initial diagnosis of MM was defined as the index date and the baseline period was defined as the 36-month period prior to the index date. Eligible patients were followed up from the index date to the date of death or the end of the study period.

### Study population

Patients aged 19 years or older who were diagnosed with MM (ICD-10: C90, C90.0) at least once during the index period were included in the study population (See supplementary methods for detailed operational definitions and codes). Patients diagnosed with MM, plasma cell leukemia (ICD-10: C90.1), or metastatic solid tumors (ICD-10: C78.x) without primary cancer (ICD-10: C00.x-C97.x, except for C77.x-C89.x) during the baseline period were excluded from the study population. The study population was further categorized into those who received stem cell transplants (SCT) and those who did not (non-SCT) during the follow-up period.

### MM drugs and line of therapy identification

**Fig. 1:**
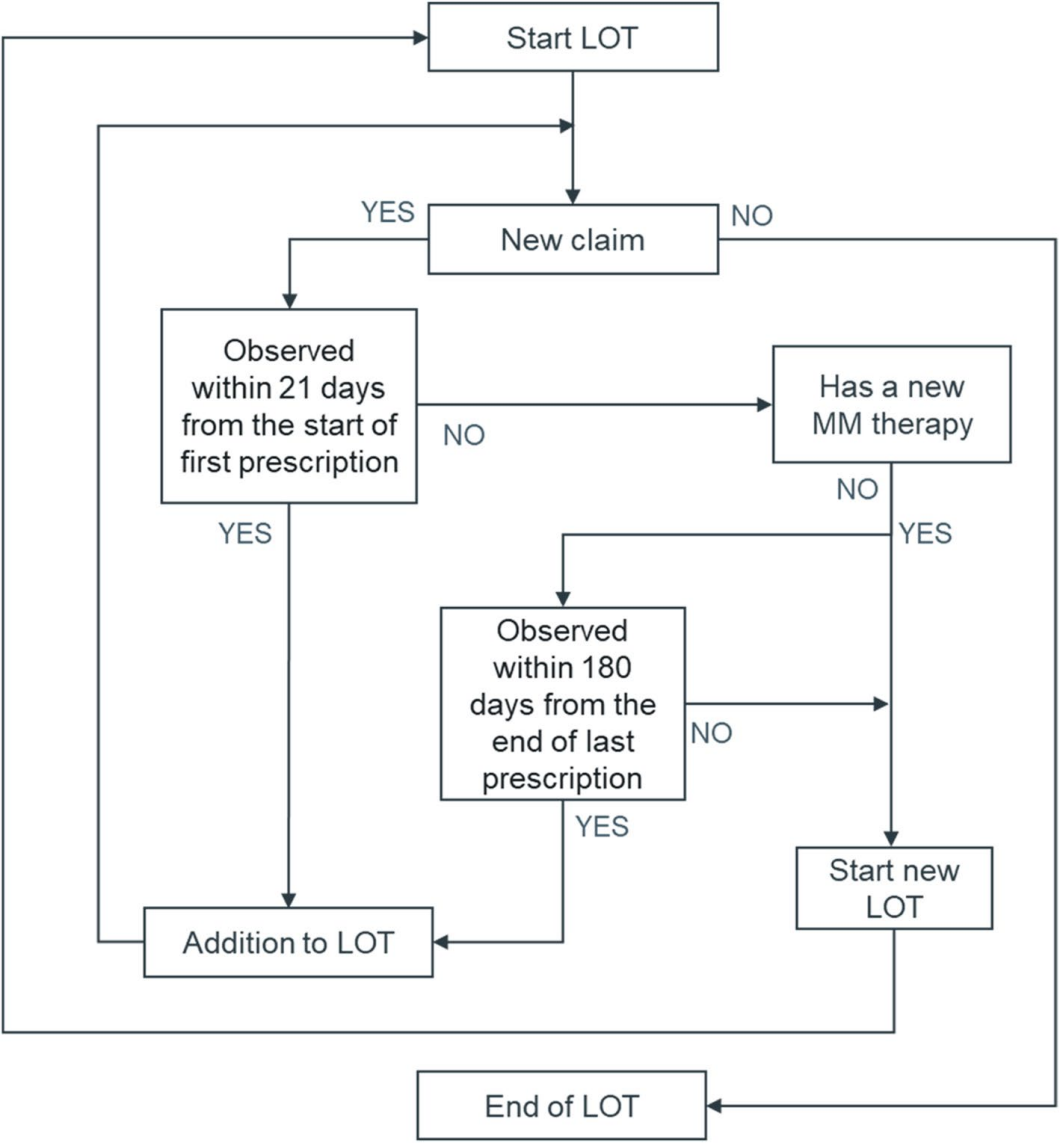
Line of therapy identification algorithm. LOT, line of therapy.

To define LOTs for MM treatment, an identification algorithm was developed based on a published, validated algorithm and real-world clinical practice (Fig. 1) [11, 12].

For each selected patient, the first LOT (LOT 1) for MM therapy was considered initiated when the patient was prescribed with any of the following MM drugs of interest during the follow-up period: bortezomib (V), melphalan (M), carfilzomib (K), thalidomide (T), lenalidomide (R), pomalidomide (P), daratumumab (D), cyclophosphamide (C), doxorubicin (A), liposomal doxorubicin (A’), vincristine (v), cisplatin (P’), and etoposide (e) (See Supplementary Methods). Subsequent LOTs were considered initiated when patients were prescribed any MM drug that was not part of the regimen identified within the first 21 days from LOT initiation, or that was prescribed 180 days from the end of the last prescription of the respective LOT. Discontinuation of LOT was defined as the last prescription before the earliest of the following: a new claim for a subsequent LOT or no further claim with MM drugs. The discontinuation date was defined as the 30th day following the start date of the last LOT prescription or the day before the initiation date of the subsequent LOT, whichever occurred first. To exclude drugs used for mobilization or conditioning, E and/or C prescribed within 30 days prior to mobilization and E, C, and/or M prescribed within 7 days prior to SCT were not considered in the LOT identification process (Supplementary Methods).

### Outcomes

Treatment patterns and outcomes were assessed as the real-world treatment sequence, treatment-free interval, treatment duration (rwTD), time to next-line treatment (rwTTNT), and overall survival (rwOS). The treatment sequence was described as the progression from LOT 1 to the subsequent LOTs or death during the follow-up period. The treatment-free interval between LOTs was defined as the duration between the LOT discontinuation and subsequent LOT initiation. The rwTD of each LOT was defined as the period from LOT initiation to LOT discontinuation, and the rwTTNT was defined as the duration from LOT initiation to the subsequent LOT initiation. Death was identified using relevant diagnostic codes (ICD-10: I46.1, R96.x, R98.x, and R99.x) and treatment result codes for death.

Healthcare resource utilization (HCRU) and costs during rwTD and rwTTNT were assessed for each LOT and reported per person per month (PPPM). All-cause HCRU included hospitalizations and outpatient medical visits. The all-cause healthcare costs included all expenses identifiable in any claims recorded during the follow-up period. MM treatment-related HCRU and costs were identified using claims with diagnostic codes for MM and MM-related comorbidities (ICD-10: MM C90, C90.0; renal failure/kidney disease N17.x-N19. x; anemia D55.x-D64.x; fractures T02.x, T08.x, T10.x, T12.x, and T14.2; bacterial disease A30.x-A59.x) during the follow-up period (Supplementary Methods). All costs were calculated in Korean won (KRW) and converted to United States dollars (USD) using the average exchange rate for 2020 (1 USD=1179.199 KRW). Additionally, costs were adjusted for the calendar year 2020 using data for the medical care component of the Consumer Price Index of South Korea [13, 14].

### Statistical analysis

All outcomes are summarized using descriptive statistics. Continuous variables were summarized as mean with standard deviation (SD), and categorical variables were summarized as frequencies and proportions (%). For comparisons, a t-test or ANOVA was used for continuous variables, and chi-square test was used for categorical variables. A Sankey diagram was plotted to visualize the progression of the LOT sequence. rwTTNT and rwOS were estimated using the Kaplan-Meier (KM) method and compared across LOTs using the log-rank test. All statistical analyses were performed using SAS® 9.4 software (SAS Institute, North Carolina, US) via SAS Enterprise Guide version 6.1, with two-sided tests and a significance level of 0.05.

## Results

### Demographic and clinical characteristics

**Fig. 2:**
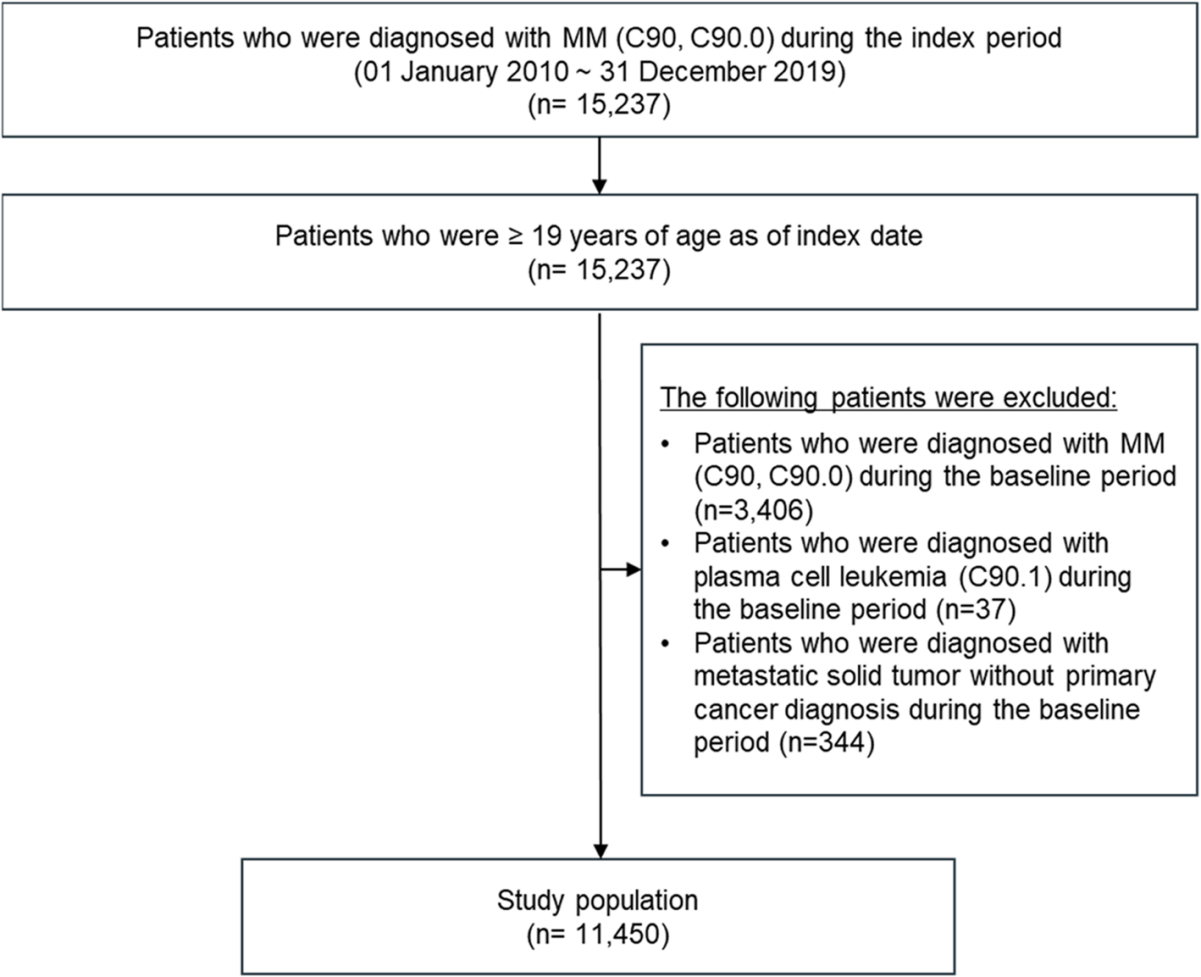
Flow chart of patient selection. MM, multiple myeloma.

A total of 15,237 patients were initially diagnosed with MM between 2010 and 2019. After excluding patients diagnosed with MM, plasma cell leukemia, or metastatic solid tumors without primary cancer during the baseline period, the database included 11,450 newly diagnosed patients with MM as the study population (Fig. 2).

The demographic and clinical characteristics of patients with MM are summarized in Table 1. The number of enrolled patients showed an increasing trend over the study period, from 873 in 2010 to 1,464 in 2019 (7.62% and 12.79%, respectively). Of the total study population, 42.40% were aged 70 years or older and 54.39% were male. The mean CCI score was 3.24 (SD 2.43), with 73.33% of the patients having a CCI score of two or higher. The most common comorbidity was chronic pulmonary disease (54.55%), followed by peptic ulcer disease (48.67%) and mild liver disease (38.47%). The total patient cohort was divided into 3,080 patients (26.9%) in the SCT group and 8,370 (73.1%) in the non-SCT group. As expected, the non-SCT group exhibited characteristics indicative of advanced frailty compared to the SCT group, including older age, a higher prevalence of comorbidities across CCI categories, and elevated CCI scores.

**Table 1:** Demographic and clinical characteristics

**Variables**	**All **(*n*=11,450)		**SCT STATUS**				
			**SCT **(*n*=3,080)		**non-SCT **(*n*=8,370)		***P*-value†**
	**N**	**%**	**N**	**%**	**N**	**%**	
**Age group**							
19-59	3,098	27.06	2,092	67.92	1,006	12.02	<0.001
60-69	3,497	30.54	984	31.95	2,513	30.02	
70+	4,855	42.40	4	0.13	4,851	57.96	
**Sex**							
Male	6,228	54.39	1,731	56.20	4,497	53.73	0.018
Female	5,222	45.61	1,349	43.80	3,873	46.27	
**Index year**							
2010	873	7.62	233	7.56	640	7.65	0.099
2011	849	7.41	205	6.66	644	7.69	
2012	1,049	9.16	282	9.16	767	9.16	
2013	1,124	9.82	298	9.68	826	9.87	
2014	1,150	10.04	290	9.42	860	10.27	
2015	1,130	9.87	289	9.38	841	10.05	
2016	1,263	11.03	379	12.31	884	10.56	
2017	1,246	10.88	327	10.62	919	10.98	
2018	1,302	11.37	371	12.05	931	11.12	
2019	1,464	12.79	406	13.18	1,058	12.64	
**CCI scores**							
Mean (SD)	3.24 (2.43)		2.22 (1.96)		3.61 (2.48)		<0.001
Median (Q1-Q3)	3.00 (1.00-5.00)		2.00 (1.00-3.00)		3.00 (2.00-5.00)		
0, 1	3,054	26.67	1,303	42.31	1,751	20.92	<0.001
2	2,079	18.16	663	21.53	1,416	16.92	
3	1,802	15.74	457	14.84	1,345	16.07	
4 +	4,515	39.43	657	21.33	3,858	46.09	
**CCI category**							
Myocardial infarction	272	2.38	33	1.07	239	2.86	<0.001
Congestive heart failure	1,454	12.70	151	4.90	1,303	15.57	<0.001
Peripheral vascular disease	2,942	25.69	488	15.84	2,454	29.32	<0.001
Cerebrovascular disease	2,059	17.98	258	8.38	1,801	21.52	<0.001
Dementia	312	2.72	19	0.62	293	3.50	<0.001
Chronic pulmonary disease	6,246	54.55	1,386	45.00	4,860	58.06	<0.001
Connective tissue disease	933	8.15	225	7.31	708	8.46	0.045
Peptic ulcer disease	5,573	48.67	1,320	42.86	4,253	50.81	<0.001
Mild liver disease	4,405	38.47	1,041	33.80	3,364	40.19	<0.001
Diabetes without chronic complication	3,779	33.00	650	21.10	3,129	37.38	<0.001
Diabetes with chronic complication	1,530	13.36	225	7.31	1,305	15.59	<0.001
Hemiplegia or paraplegia	191	1.67	18	0.58	173	2.07	<0.001
Renal disease	1,102	9.62	119	3.86	983	11.74	<0.001
Any malignancy, including lymphoma and leukemia, except malignant neoplasm of skin	1,486	12.98	242	7.86	1,244	14.86	<0.001
Moderate or severe liver disease	111	0.97	16	0.52	95	1.14	0.003
Metastatic solid tumor	25	0.22	4	0.13	21	0.25	0.219
AIDS/HIV	3	0.03	0	0.00	3	0.04	0.293
**History of other cancer**							
Yes	1,498	13.08	275	8.93	1,223	14.61	<0.001
No	9,952	86.92	2,805	91.07	7,147	85.39	
**History of anticancer therapy use**							
Yes	1,158	10.11	184	5.97	974	11.64	<0.001
No	10,292	89.89	2,896	94.03	7,396	88.36	
**SCT during follow-up period**							
Yes	3,080	26.90	3,080	100.00	0	0.00	NA
No	8,370	73.10	0	0.00	8,370	100.00	
**Other cancer during follow-up period**							
Yes	1,539	13.44	319	10.36	1,220	14.58	<0.001
No	9,911	86.56	2,761	89.64	7,150	85.42	
**Follow-up period (month)**							
Mean (SD)	42.25 (34.00)		65.89 (34.22)		67.33 (34.20)		0.045
Median (Q1-Q3)	33.26 (14.97-63.49)		62.29 (35.77-94.49)		65.58 (37.37-95.66)		

*CCI* Charlson Comorbidity Index, *SCT* Stem cell transplant, *SD* Standard deviation, *Q* Quartile, *NA* Not applicable.

^†^Derived from t-test for continuous variables and chi-square test for categorical variables

### Treatment patterns

Following the initial diagnosis of MM, 85.81% of the study population received LOT 1, 46.69% continued to receive LOT 2, 24.10% received LOT 3, 12.50% received LOT 4, and 6.19% received LOT 5. The proportion of patients who transitioned to subsequent LOTs was consistently higher in the SCT group than that in the non-SCT group across all LOTs.

**Fig. 3:**
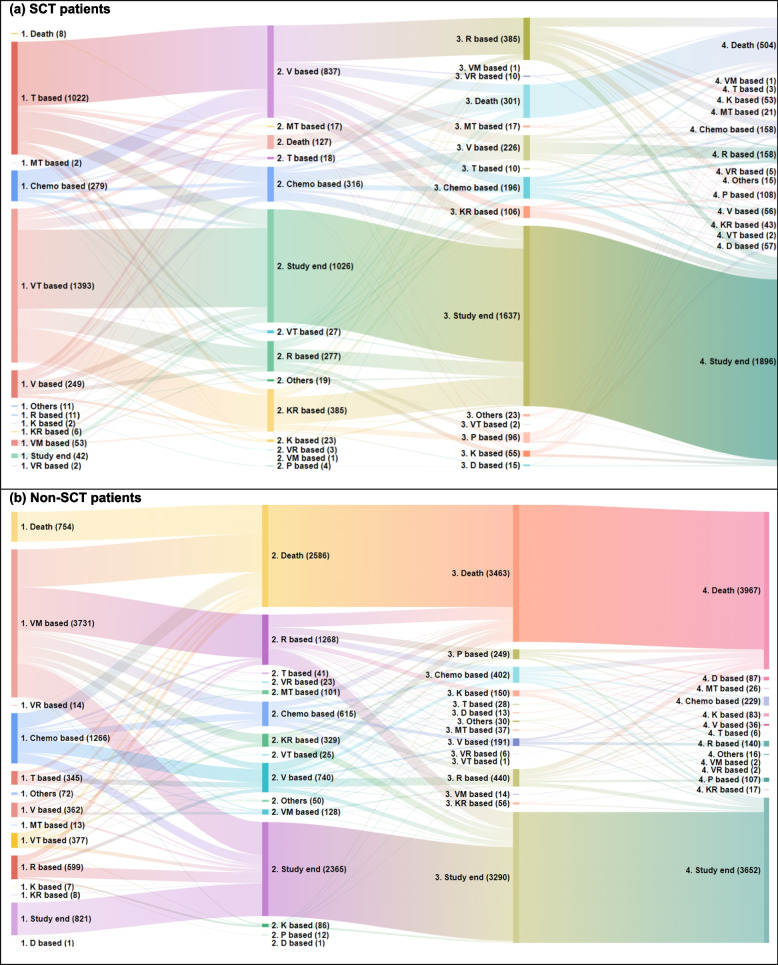
Treatment sequence from the first line of therapy to third line of therapy in patients who underwent SCT (**a**) and in patients who did not undergo SCT (**b**). SCT, Stem cell transplant; V, Bortezomib; M, Melphalan; K, Carfilzomib; T, Thalidomide; R, Lenalidomide; P, Pomalidomide; D, Daratumumab; C, Cyclophosphamide; A, Doxorubicin; A’, Liposomal doxorubicin; v, Vincristine; P’, Cisplatin; e, Etoposide. † Date of reimbursement scheme change for the SCT patients. ‡ Date of reimbursement scheme change for the non-SCT patients

In the SCT group, the most frequently received regimens were VT-based on LOT 1 (45.97%), V-based on LOT 2 (43.44%), R-based on LOT 3 (33.71%), and chemotherapy- and R-based on LOT 4 (23.24% and 23.24%, respectively; Fig. 3a, Supplementary Table 3). In the non-SCT group, the most frequently received regimens were VM-based on LOT 1 (54.91%) and LOT 2 (37.09%), and chemotherapy-based on LOT 3 (27.21%) and LOT 4 (30.49%; Fig. 3b, Supplementary Table 4).

Treatment sequence patterns have notably changed following reimbursement scheme changes in South Korea. The V and VT regimens became available for patients who received SCT in 2015, and the R regimen became available for patients who did not receive SCT in December 2017. Before the change, the most common sequences in the SCT group were the T-based regimen (11.71%) and the T-based regimen with a subsequent V-based regimen (9.44%). After this change, the most frequent sequences in the SCT group shifted to the VT-based regimen (48.11%) and the VT-based regimen with a subsequent KR-based regimen (12.11%; Figure 3a). In the non-SCT group, the most common sequences were the VM-based regimen (24.76%) and chemotherapy (11.35%), which shifted to the VM-based regimen (30.01%) and R-based regimen (22.37%), after the reimbursement scheme change (Fig. 3b). More than half of the total study cohort received only LOT 1 (39.12%) or up to LOT 2 (22.59%) and did not receive further therapies during the follow-up period, indicating that most treatment sequences ended with earlier LOTs.

The mean length of treatment-free intervals decreased with each advancing LOT after LOT 1 initiation in both the SCT and non-SCT groups, decreasing from 18.84 months (SD 19.55) to 2.94 months (SD 6.83) and from 7.50 months (SD 12.26) to 2.57 months (SD 5.29), respectively. While the mean rwTD showed a fluctuating trend in the SCT group, the mean rwTD in the non-SCT group consistently decreased with each LOT, except for LOT 1, declining from 8.78 months (SD 10.08) in LOT 2 to 6.05 months (SD 8.04) in LOT 4 (Table 2).

**Table 2:** Proportion of patients reaching each line of therapy, treatment-free intervals, and treatment durations

	**All** (*n*=11,450)		**SCT STATUS**			
			**SCT** (*n*=3,080)		**non-SCT** (*n*=8,370)	
	**mean (SD)**		**mean (SD)**		**mean (SD)**	
**Patients reaching each line (N, %)**						
LOT 1	9,825	85.81	3,030	98.38	6,795	81.18
LOT 2	5,346	46.69	1,972	64.03	3,419	40.85
LOT 3	2,759	24.10	1,142	37.08	1,617	19.32
LOT 4	1,431	12.50	680	22.08	751	8.97
LOT 5+	709	6.19	371	12.05	338	4.04
**Real-world treatment-free interval (month)**						
Initial MM diagnosis – LOT 1 initiate	1.92 (7.52)		1.57 (6.67)		2.07 (7.87)	
LOT 1 discontinue – LOT 2 initiate	11.59 (16.23)		18.84 (19.55)		7.50 (12.26)	
LOT 2 discontinue – LOT 3 initiate	6.50 (11.74)		8.64 (13.76)		5.00 (9.80)	
LOT 3 discontinue – LOT 4 initiate	4.28 (9.17)		5.26 (10.74)		3.40 (7.38)	
LOT 4 discontinue – LOT 5 initiate	2.77 (6.14)		2.94 (6.83)		2.57 (5.29)	
**Real-world treatment duration (month)**						
LOT 1 initiate – LOT 1 discontinue	7.14 (6.85)		4.50 (2.22)		8.32 (7.82)	
LOT 2 initiate – LOT 2 discontinue	8.30 (9.19)		7.47 (7.29)		8.78 (10.08)	
LOT 3 initiate – LOT 3 discontinue	7.34 (8.58)		8.15 (9.08)		6.78 (8.15)	
LOT 4 initiate – LOT 4 discontinue	6.58 (8.14)		7.16 (8.21)		6.05 (8.04)	
LOT 5 initiate – LOT 5+ discontinue*	9.33 (10.37)		9.43 (10.12)		9.23 (10.64)	

*LOT* Line of therapy, *SCT* Stem cell transplant, *SD* Standard deviation

^*^Until the earliest of end of last LOT treatment, death, and end of study period

### Treatment outcomes

**Fig. 4:**
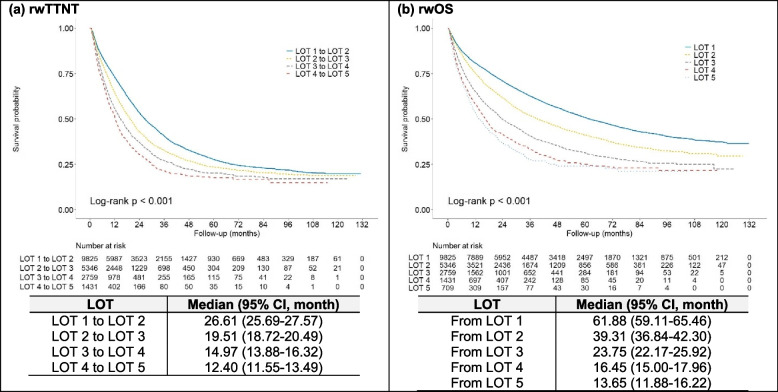
Real-world time to next-line treatment by line of therapy (**a**) and survival following the initiation of each line of therapy (**b**). rwOS, Real-world overall survival; rwTTNT, Real-world time to next-line treatment, LOT, Line of therapy

A progressive decline was observed in both rwTTNT and rwOS across successive LOTs. The KM-estimated median rwTTNT decreased from 26.61 months (95 CI: 25.69-27.57) at LOT 1 to 12.40 months (95% CI: 11.55-13.49) at LOT 4 (*P*<0.001, Fig. 4a), and the KM-estimated median rwOS decreased from 61.88 months (95% CI: 59.11-65.46) following LOT 1 to 13.65 months (95% CI: 11.88-16.22) following LOT 5 (*P*<0.001, Fig. 4b).

### Healthcare resource utilization and cost

**Fig. 5:**
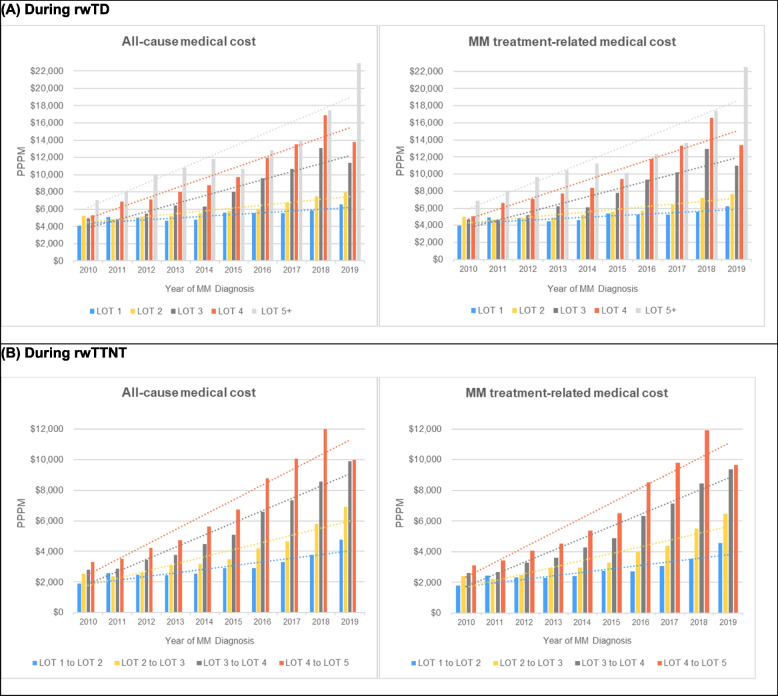
Trends of all-cause and MM treatment-related medical costs by year of MM diagnosis and LOT advancement during rwTD (**A**) and rwTTNT (**B**). MM, multiple myeloma; LOT, Line of therapy; rwTD, Real-world treatment duration; rwTTNT; Real-world time to next line treatment.

During rwTDs, all-cause costs increased steadily with the advancement of LOTs, rising from $5,365.93 PPPM in LOT 1 to $10,641.24 PPPM in LOT 5+ (*P*<0.001). Additionally, all-cause costs showed an upward trend with more recent diagnoses, escalating from $3,698 to $6,046 PPPM between 2010 and 2019 (Fig. 5a, Supplementary Table 5). Similarly, during rwTTNTs encompassing treatment-free intervals, all-cause costs, increased as the LOT progressed, rising from $3,017 to $4,820 PPPM (Fig. 5b, Supplementary Table 5). Of note, MM treatment-related costs during rwTDs and rwTTNTs consistently increased with LOT advancement and diagnosis year, constituting the majority of the all-cause costs (Fig. 5a, Fig. 5b). However, both the all-cause and MM treatment-related HCRU remained relatively stable without notable fluctuations across the treatment lines (Supplementary Table 5).

## Discussion

This large-scale observational study offers extensive insights into the real-world treatment patterns, outcomes, and economic burdens by utilizing nationwide patient-level data from South Korea. The strength of this study lies in its systematic approach to identify treatment lines and delineate the representative trends of outcomes and economic burden across LOTs throughout the disease course.

This study included 11,450 patients newly diagnosed with MM in South Korea, with the number gradually increasing from 873 in 2010 to 1,464 in 2019, which is consistent with a national report on cancer. According to the 2019 Annual Report of Cancer Statistics in Korea, there were 1,080 newly diagnosed cases of MM in 2010 and 1,831 in 2019, indicating that our study captured over 80% of patients with MM in South Korea [15]. The cohort in this study comprised 54.39% male patients, with the most common comorbidities being chronic pulmonary disease (54.55%), peptic ulcer disease (48.67%), liver disease (38.47%), and diabetes without chronic complications (33.0%). These epidemiological findings align with the results from another nationwide Korean dataset from the National Health Insurance Service, which reported 54.3% male patients, and the most frequent comorbidities were chronic pulmonary disease (44.9%), peptic ulcer disease (40.7%), diabetes without chronic complications (24.1%), and liver disease (23.8%) [16]. Therefore, we believe that our study population reasonably reflects the characteristics of patients with MM in South Korea, which may enhance the generalizability of our findings to a broader MM patient population in the country and potentially to similar populations.

Based on the comprehensively defined LOT identification algorithm, this study generated timely real-world evidence on treatment patterns and outcomes, enabling physicians to make realistic estimates and, thus, optimal, well-informed choices throughout the patients’ treatment journey. As expected, the rwTDs and treatment-free intervals in this study showed a decreasing trend with LOT advancement from the second line onward, aligning with a prior multi-country, retrospective, real-world study. In that study, the reported mean rwTDs were 8 months (95% CI: 7.74-8.26), 9 months (95% CI: 8.64-9.36), 8 months (95% CI: 7.63-8.37), and 6 months (95% CI: 5.5-6.5) at LOT 1, LOT 2, LOT 3, and LOT 4, respectively [17]. Moreover, reflective of real-world treatment patterns, since the Vd (bortezomib and dexamethasone) and VTd (bortezomib, thalidomide, and dexamethasone) regimens became available by the reimbursement scheme change in October 2015, the majority of patients who received SCT during the follow-up period in this study initiated their first-line treatment with regimens that included VT (65.63%). Similarly, following the reimbursement scheme change in December 2017, which made the Rd (lenalidomide and dexamethasone) regimen available for patients who did not receive SCT, the treatment sequence of the VM-based regimen with subsequent R-based regimens became the second most common sequence for this population [18]. With the study period spanning the introduction of several novel agents and relevant reimbursement scheme changes for MM treatment, our findings describe and confirm the shifts in treatment patterns in real-world practice, likely driven by the evolving and expanding landscape of MM therapies.

In terms of treatment outcomes, we observed a gradual reduction in rwTTNT and rwOS with each successive LOT, showing distinctive treatment outcomes across the LOTs. This trend is further evidenced by other large-scale studies: a multi-center, retrospective cohort study involving 30 clinics in Latin American countries reported that the median progression-free survival decreased from 15.0 months and 31.1 months to 10.9 months and 9.5 months following LOT 1 initiation in the patients who received SCT and who did not, respectively [19]. An observational chart review involving seven countries showed that median LOT duration, interval, and time to progression decreased with increasing lines of therapy [17].

Following the initial diagnosis of MM, patients generally show relatively favorable responses to their first treatment approach. However, MM frequently relapses, and intervals between treatments become increasingly shorter as the disease becomes less responsive to treatment in later treatment lines. This progression has results in an increased economic burden [20]. Our findings suggest that the economic burden, as highlighted in this study , as quantified through medical costs, increased considerably with LOT progression despite healthcare resource utilization remaining consistent across different LOTs. The impact of relapsed refractory MM on advanced treatment lines is substantial and is becoming a critical concern [21]. Previous retrospective cohort studies using Truven Analytics MedStat MarketScan® Commercial Claims and Encounters and Medicare and Coordination of Benefits data have shown that the total annual healthcare costs were higher with disease progression, defined as advancement to the next LOT, compared to without progression [22, 23]. Furthermore, our study demonstrated that medical costs increased concurrently over the study period, with a paradigm shift in MM management [24]. Given the rising drug prices over time and in advanced LOTs, the correlation between increasing LOTs and medical costs may seem intuitive. However, our findings provide a robust empirical quantification of this relationship using large-scale data, which is crucial for validating assumptions and guiding healthcare policies and resource allocation decisions. These trends underscore the significance of accelerating patients’ access to advanced therapies from earlier treatment lines, which may further facilitate more effective allocation of limited resources [23].

While the LOT identification algorithm used in this study was developed to account for real-world local practices, it should be noted that not all scenarios could have been covered by the algorithm. There is a possibility that drug misclassification into regimens and LOT sequences could arise. For instance, because of the nature of the claims data, MM drugs not covered by healthcare insurance were not considered in this study. Most regimens used for conditioning and mobilization are not covered by healthcare insurance and are thus not considered in the LOT identification algorithm. In addition, the claims data lacks detailed clinical information, such as disease progression markers or reasons for treatment discontinuation, which could be due to clinical decisions, safety events, or patient needs. Consequently, the applicability of the LOT algorithm may be compromised in certain cases, especially when treatment breaks occur because of side effects or other patient-specific factors not captured in the data. To minimize potential misclassifications and discrepancies, the algorithm was developed based on a validated algorithm and refined through extensive consultations with local clinicians with medical adjudication accompanying the LOT identification process. Additionally, as the information on death in the HIRA database is limited to in-hospital deaths, it is likely that some out-of-hospital death events were not captured in the the study. However, overall trends and median OS aligned with those of a previous study that similarly analyzed MM outcomes by LOT from 2011 to 2019 using nationwide electronic health records in the USA. This study reported a median OS of 60 months for patients treated with the first-line treatment, which decreased to 48, 36, 29, and 23 months for LOT 2, LOT 3, LOT 4, and LOT 5+, respectively [25]. The use of large-scale real-world data has limitations, including the potential introduction of certain constraints inherent in larger datasets, and the findings should be interpreted with an appropriate level of caution. However, we believe that these real-world data offer valuable opportunities to capture a broad spectrum of data-driven patient experiences and treatment patterns, which may not be feasible in smaller, controlled studies.

This large population-based study demonstrated that the advancement of LOT is associated with disease progression, leading to increased medical costs for MM. As patients with MM live longer and progress through more lines of therapy, there is a growing interest in exploring better treatment options to enhance outcomes and effectively manage resources in advanced stages. A strategic approach to advanced treatment is warranted to improve patient survival and manage the financial burden more efficiently.

## Supplementary Information

Supplementary Material 1.

## Data Availability

The data supporting the findings of this study are available from the Health Insurance Review and Assessment (HIRA) Service of South Korea. These data are not publicly available due to their containing information that could compromise the privacy of research participants. Access to the data is available through the HIRA Service (https://www.hira.or.kr) with appropriate permissions. Researchers interested in accessing the data can apply through the HIRA website.
