# Supplementary material for: Real-world treatment patterns, outcomes, and economic costs by lines of therapy in patients with newly diagnosed multiple myeloma: a nationwide population-based cohort study in South Korea
Source: Blood Res. 2025 Apr 15;60(1):26. doi: 10.1007/s44313-025-00069-3 (PMC12000491; doi:10.1007/s44313-025-00069-3)
Supplement: Supplementary file 1 — Supplementary Material 1. [file 44313_2025_69_MOESM1_ESM.docx]

# SUPPLEMENTARY METHODS

MM diagnosis was defined as following:

1. Defined as ≥ 1 outpatient or inpatient claim with the International Classification of Diseases 10th Edition (ICD-10) code for MM recorded as primary diagnosis with special benefit code for registered cancer patients, followed by ≥ 1 outpatient or inpatient claim with ICD-10 code for MM recorded as primary diagnosis during the index period; AND
   - ICD-10 code for MM: C90, C90.0
   - Special benefit code for registered cancer patients: V193, V194, V027
2. ≥ 1 outpatient or inpatient claim with procedure code for BM transplant within the 36-month period prior to or after the date of first MM diagnosis during the index period
   - Procedure code for bone marrow transplantation: B1111, B1112, B1113, B1114, B1115, B1117, C5520, C8031, C8520, D0831, D0832, D0833, HC201, HC202, HC203

MM drugs were identified using the Heath Insurance Review and Assessment (HIRA) drug codes corresponding to the following World Health Organization-Anatomical Therapeutic Chemical (WHO-ATC) codes (Supplementary Table 1):

1. WHO-ATC code for bortezomib (V): L01XX32
2. WHO-ATC code for melphalan (M): L01AA03
3. WHO-ATC code for carfilzomib (K): L01XX45, L01XG02
4. WHO-ATC code for thalidomide (T): L04AX02
5. WHO-ATC code for lenalidomide (R): L04AX04
6. WHO-ATC code for pomalidomide (P): L04AX06
7. WHO-ATC code for daratumumab (D): L01XC24
8. WHO-ATC code for doxorubicin (A): L01DB01
9. WHO-ATC code for liposomal doxorubicin (A’): L01DB01
10. WHO-ATC code for cyclophosphamide (C): L01AA01
11. WHO-ATC code for vincristine (v): L01CA02
12. WHO-ATC code for cisplatin (P’): L04AX06
13. WHO-ATC code for etoposide (e): L01CB01

Supplementary Table 1. List of MM drugs and relevant HIRA molecule codes

| **Drug** | **HIRA molecule code** |
| --- | --- |
| Bortezomib | 463301BIJ |
|  | 463302BIJ |
|  | 463303BIJ |
| Melphalan | 189901ATB |
| Carfilzomib | 647801BIJ |
|  | 647802BIJ |
| Thalidomide | 485701ACH |
|  | 485702ACH |
| Lenalidomide | 588201ACH |
|  | 588201ATB |
|  | 588202ACH |
|  | 588202ATB |
|  | 588203ACH |
|  | 588203ATB |
|  | 588204ACH |
|  | 588204ATB |
|  | 588205ACH |
|  | 588205ATB |
|  | 588206ACH |
|  | 588206ATB |
|  | 588207ACH |
|  | 588207ATB |
| Pomalidomide | 628001ACH |
|  | 628002ACH |
|  | 628003ACH |
|  | 628004ACH |
| Daratumumab | 667101BIJ |
|  | 667102BIJ |
| Cyclophosphamide | 139001ATB |
| Doxorubicin hydrochloride | 149401BIJ |
|  | 149402BIJ |
|  | 149404BIJ |
|  | 149405BIJ |
|  | 149430BIJ |
|  | 149432BIJ |
|  | 149433BIJ |
|  | 149434BIJ |
| Liposomal doxorubicin hydrochloride | 149403BIJ |
|  | 149406BIJ |
|  | 149431BIJ |
|  | 149435BIJ |
| Vincristine sulfate | 248001BIJ |
|  | 248002BIJ |
|  | 248003BIJ |
|  | 248030BIJ |
|  | 248031BIJ |
|  | 248032BIJ |
| Cisplatin | 134501BIJ |
|  | 134502BIJ |
|  | 134503BIJ |
|  | 134530BIJ |
|  | 134531BIJ |
|  | 134532BIJ |
|  | 134533BIJ |
|  | 134534BIJ |
| Etoposide | 157101BIJ |
|  | 157102ACH |
|  | 157103BIJ |
|  | 157104ACH |
|  | 157104BIJ |
|  | 157105BIJ |
|  | 157106BIJ |
|  | 157107BIJ |
|  | 157108BIJ |
|  | 157130BIJ |
|  | 157131BIJ |
|  | 157132BIJ |
|  | 157133BIJ |
|  | 157134BIJ |
|  | 157135BIJ |
|  | 157136BIJ |

MM drugs were categorized at class level as following:

1. D-based regimens: all regimens that include D
2. VR-based regimens: (among the remaining regimens) regimens that include VR
3. VT-based regimens: (among the remaining regimens) regimens that include VT
4. LR-based regimens: (among the remaining regimens) regimens that include KR
5. MT-based regimens: (among the remaining regimens) regimens that include MT
6. VM-based regimens: (among the remaining regimens) regimens that include VM
7. Chemotherapy-based regimens: (among the remaining regimens) regimens that include M, V’A, or C
8. V-based regimens: (among the remaining regimens) regimens that include V
9. R-based regimens: (among the remaining regimens) regimens that include R
10. T-based regimens: (among the remaining regimens) regimens that include T
11. K-based regimens: (among the remaining regimens) regimens that include K
12. P-based regimens: (among the remaining regimens) regimens that include P
13. Others regimens: remaining regimens that were not categorized as 1)-12)

Following MM drugs used during the mobilization and/or conditioning periods were not considered (i.e., ignored) for LOT identification:

1. E and/or C that were prescribed during the 30-day period prior to and including the date of mobilization.
   - Date of mobilization was defined as the starting date of the inpatient claim with the procedure code recorded for mobilization the follow-up period, including the index date
   - Procedure code for mobilization: X5011-4, X5020-4, X5031-3, X5041-2, X5061-4, X5111-5, X5120
2. E, C, and/or M that were prescribed during the 7-day period prior to and including the date of SCT
   - Date of SCT was defined as the starting date of inpatient claim with procedure code recorded for autologous SCT or allogeneic SCT during the follow-up period, including the index date
   - Procedure code for ASCT/allogeneic SCT: X5051, X5131-6

Clinical characteristics during the baseline period were defined as following:

1. Charlson Comorbidity Index (CCI)
   - Defined using the pre-defined scores and relevant ICD-10 codes recorded as primary - 4th secondary diagnosis during the 36-month period prior to the index date, excluding the latest 1 month prior to the index date (Supplementary Table 2)

Supplementary Table 2. Charlson Comorbidity Index

| **Comorbidity** | **ICD-10 code** | **Score** |
| --- | --- | --- |
| Myocardial infarction | I21.x, I22.x, I25.2 | 1 |
| Congestive heart failure | I09.9, I11.0, I13.0, I13.2, I25.5, I42.0, I42.5–I42.9, I43.x, I50.x, P29.0 | 1 |
| Peripheral vascular disease | I70.x, I71.x, I73.1, I73.8, I73.9, I77.1, I79.0, I79.2, K55.1, K55.8, K55.9, Z95.8, Z95.9 | 1 |
| Cerebrovascular disease | G45.x, G46.x, H34.0, I60.x–I69.x | 1 |
| Dementia | F00.x–F03.x, F05.1, G30.x, G31.1 | 1 |
| Chronic pulmonary disease | I27.8, I27.9, J40.x–J47.x, J60.x–J67.x, J68.4, J70.1, J70.3 | 1 |
| Connective tissue disease | M05.x, M06.x, M31.5, M32.x–M34.x, M35.1, M35.3, M36.0 | 1 |
| Peptic ulcer disease | K25.x–K28.x | 1 |
| Mild liver disease | B18.x, K70.0–K70.3, K70.9, K71.3–K71.5, K71.7, K73.x, K74.x, K76.0, K76.2–K76.4, K76.8, K76.9, Z94.4 | 1 |
| Diabetes without chronic complication | E10.0, E10.1, E10.6, E10.8, E10.9, E11.0, E11.1, E11.6, E11.8, E11.9, E12.0, E12.1, E12.6, E12.8, E12.9, E13.0, E13.1, E13.6, E13.8, E13.9, E14.0, E14.1, E14.6, E14.8, E14.9 | 1 |
| Diabetes with chronic complication | E10.2–E10.5, E10.7, E11.2–E11.5, E11.7, E12.2–E12.5, E12.7, E13.2–E13.5, E13.7, E14.2–E14.5, E14.7 | 2 |
| Hemiplegia or paraplegia | G04.1, G11.4, G80.1, G80.2, G81.x, G82.x, G83.0–G83.4, G83.9 | 2 |
| Renal disease | I12.0, I13.1, N03.2–N03.7, N05.2–N05.7, N18.x, N19.x, N25.0, Z49.0–Z49.2, Z94.0, Z99.2 | 2 |
| Any malignancy, including lymphoma and leukemia, except malignant neoplasm of skin | C00.x–C26.x, C30.x–C34.x, C37.x–C41.x, C43.x, C45.x–C58.x, C60.x–C76.x, C81.x–C85.x, C88.x,C91.x–C97.x | 2 |
| Moderate or severe liver disease | I85.0, I85.9, I86.4, I98.2, K70.4, K71.1, K72.1, K72.9, K76.5, K76.6, K76.7 | 3 |
| Metastatic solid tumor (with primary cancer) | C78.x | 6 |
| AIDS/HIV | B20.x–B22.x, B24.x | 6 |

ICD, International Classification of Diseases 10th Edition

1. History of other cancer (except for MM-related cancer and metastatic solid tumor without primary cancer)
   - Defined as ≥ 1 outpatient or inpatient claim with ICD-10 code for other cancer recorded as primary diagnosis during the 36-month period prior to and excluding the index date
   - ICD-10 code for other cancers: C00.x -C97.x except for C40.x, C41.x, C76.x, C77.x, C79.x, C80.x, C90.x, C96.x, C97.x
2. History of anticancer therapy use
   - Defined as ≥ 1 outpatient or inpatient claim with prescription for anticancer therapy during the 36-month period prior to and excluding the index date
   - WHO-ATC code for anticancer therapy: L01-L04, G03BA03

Clinical characteristics during the follow-up period were defined as following:

1. SCT status during the follow-up period
   - Defined as ≥ 1 outpatient or inpatient claim with procedure code recorded for ASCT/allo-SCT during the follow-up period
   - Categorized as initial SCT, delayed SCT, tandem SCT, and salvage SCT
   - Procedure code for SCT: X5051, X5131-6
2. Other cancer status during the follow-up period (MM-related cancers and metastatic solid tumor without primary cancer)
   - Defined as ≥ 1 outpatient or inpatient claim with ICD-10 code for other cancer recorded as primary diagnosis during the follow-up period
   - ICD-10 code for other cancers: C00.x -C97.x except for C40.x, C41.x, C76.x, C77.x, C79.x, C80.x, C90.x, C96.x, C97.x

MM-related HCRU and costs during the follow-up period were defined using the outpatient or inpatient claims with diagnosis recorded for MM or MM-related comorbidities (renal failure/kidney disease, anaemia, fractures, and bacterial disease), defined as following:

1. MM
   - Defined as ≥ 1 outpatient or inpatient claim with ICD-10 code for MM recorded as primary - 1st secondary diagnosis during the follow-up period
   - ICD-10 code for MM: C90, C90.0
2. Renal failure/kidney disease
   - Defined as ≥ 1 outpatient or inpatient claim with ICD-10 code for renal failure/kidney disease recorded as primary - 1st secondary diagnosis during the follow-up period
   - ICD-10 code for renal failure/kidney disease: N17.xx, N18.xx, N19.xx
3. Anaemia
   - Defined as ≥ 1 outpatient or inpatient claim with ICD-10 code for anaemia recorded as primary - 1st secondary diagnosis during the follow-up period
   - ICD-10 code for anaemia: D55.xx-D59.xx, D60.xx-D64.xx
4. Fractures
   - Defined as ≥ 1 outpatient or inpatient claim with ICD-10 code for fractures recorded as primary - 1st secondary diagnosis during the follow-up period
   - ICD-10 code for fractures: T02.xx, T08.xx, T10.xx, T12.xx, T14.2
5. Bacterial disease
   - Defined as ≥ 1 outpatient or inpatient claim with ICD-10 code for bacterial disease recorded as primary - 1st secondary diagnosis during the follow-up period
   - ICD-10 code for bacterial disease: A30.xx-A49.xx

# SUPPMENTARY RESULTS

**Supplementary Table 3.** Treatment sequence from the first line of therapy to third line of therapy at class level in patients who underwent SCT

| **Before 2015.10.1†** (n=1,494) | **Sequence** | **N** | **(%)** |
| --- | --- | --- | --- |
|  | LOT1(T-based) | 175 | 11.71 |
|  | LOT1(T-based) - 2(V-based) | 141 | 9.44 |
|  | LOT1(T-based) - 2(V-based) - 3(R-based) | 102 | 6.83 |
|  | LOT1(T-based) - 2(KR-based) | 43 | 2.88 |
|  | LOT1(T-based) - 2(V-based) - 3(R-based) - 4(P-based) | 42 | 2.81 |
|  | LOT1(T-based) - 2(V-based) - 3(V-based) - 4(R-based) | 42 | 2.81 |
|  | LOT1(Chemo-based) | 40 | 2.68 |
|  | LOT1(T-based) - 2(V-based) - 4(R-based) - 4(Chemo-based) | 38 | 2.54 |
|  | LOT1(V-based) | 38 | 2.54 |
|  | LOT1(Chemo-based) - 2(V-based) | 37 | 2.48 |
|  | Other sequences | 796 | 53.28 |
| **After 2015.10.1†** (n=1,536) | **Sequence** | **N** | **(%)** |
|  | LOT1(VT-based) | 739 | 48.11 |
|  | LOT1(VT-based) - 2(KR-based) | 186 | 12.11 |
|  | LOT1(VT-based) - 2(R-based) | 86 | 5.60 |
|  | LOT1(VT-based) - 2(Chemo-based) | 50 | 3.26 |
|  | LOT1(V-based) | 48 | 3.13 |
|  | LOT1(VT-based) - 2(KR-based) - 3(P-based) | 25 | 1.63 |
|  | LOT1(VM-based) | 22 | 1.43 |
|  | LOT1(VT-based) - 2(KR-based) - 3(P-based) - 4(D-based) | 17 | 1.11 |
|  | LOT1(VT-based) - 2(KR-based) - 3(Chemo-based) | 16 | 1.04 |
|  | LOT1(V-based) - 2(KR-based) | 14 | 0.91 |
|  | Other sequences | 333 | 21.68 |

**Supplementary Table 4.** Treatment sequence from the first line of therapy to third line of therapy at class level in patients who did not undergo SCT

| **Before**  **2017.12.1‡** (n=5,092) | **Sequence** | **N** | **(%)** |
| --- | --- | --- | --- |
|  | LOT1(VM-based) | 1,261 | 24.76 |
|  | LOT1(Chemo-based) | 578 | 11.35 |
|  | LOT1(VM-based) - 2(R-based) | 506 | 9.94 |
|  | LOT1(Chemo-based) - 2(V-based) | 157 | 3.08 |
|  | LOT1(V-based) | 130 | 2.55 |
|  | LOT1(T-based) | 129 | 2.53 |
|  | LOT1(VM-based) - 2(Chemo-based) | 111 | 2.18 |
|  | LOT1(VM-based) - 2(KR-based) | 93 | 1.83 |
|  | LOT1(VT-based) | 84 | 1.65 |
|  | LOT1(Chemo-based)- 2(Chemo-based) | 82 | 1.61 |
|  | Other sequences | 1,961 | 38.51 |
| **After**  **2017.12.1‡** (n=1,703) | **Sequence** | **N** | **(%)** |
|  | LOT1(VM-based) | 511 | 30.01 |
|  | LOT1(R-based) | 381 | 22.37 |
|  | LOT1(VM-based) - 2(R-based) | 116 | 6.81 |
|  | LOT1(VT-based) | 101 | 5.93 |
|  | LOT1(VM-based) - 2(KR-based) | 86 | 5.05 |
|  | LOT1(R-based) - 2(V-based) | 45 | 2.64 |
|  | LOT1(Chemo-based) | 43 | 2.52 |
|  | LOT1(VT-based) - 2(KR-based) | 43 | 2.52 |
|  | LOT1(V-based) | 35 | 2.06 |
|  | LOT1(R-based) - 2(K-based) | 28 | 1.64 |
|  | Other sequences | 314 | 18.44 |

**Supplementary Table 5.** All-cause and MM treatment health care resource utilization and cost of MM patients

| **Variables** | | **rwTD†** | | | | | | | | | | | | |
| --- | --- | --- | --- | --- | --- | --- | --- | --- | --- | --- | --- | --- | --- | --- |
|  |  | **LOT 1** (n=9,825) | | **LOT 2** (n=5,346) | | **LOT 3** (n=2,759) | | **LOT 4** (n=1,431) | | **LOT 5+*** (n=709) | | ***P*-value**‡ | |  |
|  |  | **mean (SD)** | | **mean (SD)** | | **mean (SD)** | | **mean (SD)** | | **mean (SD)** | |  |  |  |
| **All-cause,**  **patient with event (N, %)** | | 9,825 | 100.00% | 5,344 | 99.96% | 2,759 | 100.00% | 1,431 | 100.00% | 709 | 100.00% |  |  |  |
| Total, visits, PPPM | | 5.18 (3.20) | | 5.53 (3.56) | | 5.34 (3.58) | | 5.26 (3.57) | | 5.19 (3.66) | | <0.001 | |  |
|  | *Inpatient visits, PPPM* | 0.70 (0.67) | | 0.70 (0.70) | | 0.77 (0.75) | | 0.86 (0.86) | | 0.82 (0.90) | | <0.001 | |  |
|  | *Outpatient visits, PPPM* | 4.87 (3.23) | | 5.36 (3.58) | | 5.19 (3.59) | | 5.05 (3.57) | | 4.94 (3.54) | | <0.001 | |  |
|  | *Length of stay, days, per admission* | 16.89 (18.81) | | 16.28 (32.37) | | 16.82 (24.42) | | 15.64 (16.91) | | 15.65 (14.83) | | 0.282 | |  |
| Total, PPPM, USD | | 5,365.93 (5,890.22) | | 5,929.50 (6,434.81) | | 7,382.92 (7,649.37) | | 8,864.79 (8,961.86) | | 10,641.24 (9,223.66) | | <0.001 | |  |
|  | *Inpatient, PPPM, USD* | 4,462.82 (6,479.27) | | 4,957.93 (7,735.12) | | 6,387.63 (9,090.07) | | 7,643.10 (10,529.26) | | 8,115.48 (10,318.46) | | <0.001 | |  |
|  | *Outpatient, PPPM, USD* | 1,548.77 (1,240.42) | | 2,602.62 (2,048.28) | | 3,232.94 (2,735.71) | | 3,624.56 (3,151.44) | | 3,801.03 (3,040.97) | | <0.001 | |  |
|  | *Pharmacy, PPPM, USD* | 93.29 (124.72) | | 120.39 (321.54) | | 123.15 (410.79) | | 104.84 (344.87) | | 101.34 (282.07) | | <0.001 | |  |
| **MM-related,**  **patient with event (N, %)** | | 9,725 | 98.98% | 5,286 | 98.88% | 2,740 | 99.31% | 1,414 | 98.81% | 635 | 89.56% |  |  |  |
| Total visits, PPPM | | 3.75 (2.50) | | 3.99 (2.86) | | 3.82 (2.91) | | 3.82 (2.87) | | 3.74 (2.90) | | <0.001 | |  |
|  | *Inpatient visits, PPPM* | 0.67 (0.64) | | 0.69 (0.69) | | 0.77 (0.74) | | 0.86 (0.86) | | 0.36 (0.34) | | <0.001 | |  |
|  | *Outpatient visits, PPPM* | 3.47 (2.50) | | 3.79 (2.89) | | 3.62 (2.92) | | 3.57 (2.87) | | 3.62 (2.89) | | <0.001 | |  |
|  | *Length of stay, days, per admission* | 17.36 (19.40) | | 17.07 (33.84) | | 17.21 (22.96) | | 15.99 (17.23) | | 15.85 (16.05) | | 0.305 | |  |
| Total, PPPM, USD | | 5,133.21 (5,855.72) | | 5,646.38 (6,316.29) | | 7,143.12 (7,625.48) | | 8,615.17 (8,991.75) | | 10,307.63 (9,175.62) | | <0.001 | |  |
|  | *Inpatient, PPPM, USD* | 4,417.31 (6,473.21) | | 5,027.14 (7,736.30) | | 6,629.07 (9,220.20) | | 7,817.91 (10,675.64) | | 8,002.46 (10,295.34) | | <0.001 | |  |
|  | *Outpatient, PPPM, USD* | 1,510.10 (1,142.32) | | 2,560.25 (2,043.50) | | 3,182.49 (2,718.31) | | 3,587.69 (3,142.38) | | 3,783.51 (3,022.37) | | <0.001 | |  |
|  | *Pharmacy, PPPM, USD* | 60.00 (114.63) | | 97.99 (334.71) | | 111.82 (475.89) | | 76.43 (169.04) | | 91.60 (338.59) | | <0.001 | |  |
| **Variables** | | **rwTTNT†** | | | | | | | | | | | | |
|  |  | **Initial diagnosis -  LOT 1 initiate** (n=9,825) | | **LOT 1 initiate - LOT 2 initiate** (n=5,346) | | **LOT 2 initiate - LOT 3 initiate** (n=2,759) | | **LOT 3 initiate - LOT 4 initiate** (n=1,431) | | **LOT 4 initiate - LOT 5 initiate** (n=709) | | ***P*-value**‡ | |  |
|  |  | **mean (SD)** | | **mean (SD)** | | **mean (SD)** | | **mean (SD)** | | **mean (SD)** | |  |  |  |
| **All-cause,**  **patient with event (N, %)** | | 6,150 | 62.60% | 5,346 | 100.00% | 2,757 | 100.00% | 1,431 | 100.00% | 709 | 100.00% |  |  |  |
| Total visits, PPPM | | 8.91 (8.64) | | 4.89 (2.97) | | 5.43 (3.43) | | 5.49 (3.43) | | 5.42 (3.57) | | <0.001 | |  |
|  | *Inpatient, PPPM* | 3.01 (4.48) | | 0.38 (0.42) | | 0.42 (0.50) | | 0.47 (0.51) | | 0.55 (0.58) | | <0.001 | |  |
|  | *Outpatient visits, PPPM* | 8.52 (8.48) | | 4.62 (2.97) | | 5.22 (3.43) | | 5.28 (3.43) | | 5.14 (3.58) | | <0.001 | |  |
|  | *Length of stay, days, per admission* | 10.88 (11.96) | | 16.85 (18.35) | | 16.27 (36.27) | | 16.27 (26.42) | | 16.19 (28.81) | | <0.001 | |  |
| Total, PPPM, USD | | 6,067.31 (7,392.86) | | 2,893.51 (2,643.02) | | 3,480.29 (3,202.87) | | 4,531.06 (3,770.48) | | 5,567.92 (4,643.67) | | <0.001 | |  |
|  | *Inpatient visits, PPPM, USD* | 7,170.19 (8,011.25) | | 1,913.82 (2,711.47) | | 2,028.22 (3,357.78) | | 2,645.43 (3,966.01) | | 3,286.85 (4,769.10) | | <0.001 | |  |
|  | *Outpatient, PPPM, USD* | 1711.96 (3362.36) | | 1134.1 (940.40) | | 2024.06 (1811.59) | | 2,781.36 (2,372.11) | | 3,286.76 (2,999.97) | | <0.001 | |  |
|  | *Pharmacy, PPPM, USD* | 120.43 (404.28) | | 80.38 (105.85) | | 97.46 (252.40) | | 97.63 (306.00) | | 97.30 (389.10) | | <0.001 | |  |
| **MM-related,**  **patient with event (N, %)** | | 6,142 | 62.51% | 5,346 | 100.00% | 2,759 | 100.00% | 1,431 | 100.00% | 709 | 100.00% |  |  |  |
| Total, visits, PPPM | | 6.42 (6.74) | | 3.16 (2.38) | | 3.62 (2.73) | | 3.79 (2.92) | | 3.77 (2.76) | | <0.001 | |  |
|  | *Inpatient visits, PPPM* | 3.05 (4.49) | | 0.36 (0.41) | | 0.41 (0.51) | | 0.47 (0.52) | | 0.54 (0.59) | | <0.001 | |  |
|  | *Outpatient visits, PPPM* | 6.08 (6.74) | | 2.91 (2.34) | | 3.42 (2.72) | | 3.58 (2.90) | | 3.51 (2.73) | | <0.001 | |  |
|  | *Length of stay, days, per admission* | 11.08 (12.38) | | 17.47 (18.79) | | 17.49 (38.58) | | 16.41 (23.25) | | 16.86 (29.78) | | <0.001 | |  |
| Total, PPPM, USD | | 5,741.78 (7,389.51) | | 2,725.33 (2,650.08) | | 3,287.54 (3,190.13) | | 4,338.79 (3,764.41) | | 5,364.34 (4,624.64) | | <0.001 | |  |
|  | *Inpatient, PPPM, USD* | 7,365.65 (8,116.19) | | 1,894.40 (2,719.60) | | 2,082.73 (3,421.44) | | 2,720.08 (4,022.32) | | 3,349.67 (4,838.31) | | <0.001 | |  |
|  | *Outpatient, PPPM, USD* | 1,657.27 (3,436.44) | | 1,075.81 (936.21) | | 1,955.24 (1,807.78) | | 2,707.69 (2,358.64) | | 3,225.64 (2,990.42) | | <0.001 | |  |
|  | *Pharmacy, PPPM, USD* | 60.83 (151.14) | | 45.87 (97.51) | | 66.22 (252.45) | | 78.65 (348.18) | | 67.16 (155.58) | | <0.001 | |  |
| rwTD, Real-world treatment duration; rwTTNT, Real-world time to next treatment; LOT, Line of therapy; MM, Multiple myeloma; PPPM, Per person per month; USD, United States Dollar; SD, Standard deviation.  † rwTD and rwTTND were calculated descriptively for each patient.  ‡ Derived from ANOVA test for continuous variables.  * Until the earliest of end of last LOT treatment, death, and end of study period | | | | | | | | | | | | | |  |
